# Time as an indispensable causal dimension in cardiovascular diseases: toward a four-dimensional biology

**DOI:** 10.3389/fcvm.2026.1952188

**Published:** 2026-10-16

**Authors:** Guido Caluori, Sebastien Chaigne

**Affiliations:** 1 IHU Liryc, INSERM, U1045, CRCTB, University of Bordeaux, Bordeaux, France; 2 CHU de Bordeaux, Cardiology, INSERM, U1045, CRCTB, Bordeaux, France

**Keywords:** atrial fibrillation, cardiovascular disease, heart failure, precision cardiology, systems biology, temporal biology, tipping point

## 1 Introduction

Heart failure (HF) exhibits distinctive event- and time-dependent patterns in incidence and progression dynamics. Over time, the heart first undergoes (mal)adaptive cellular, tissue and molecular remodelling (1, 2). These alterations precede clinical symptoms and diagnosis (3, 4) and reflect age-related changes in cardiovascular structure and function that lower the threshold for overt disease (4, 5). This subclinical process is followed by incremental cardiac remodelling, including cardiac hypertrophy (6, 7), myocardial stiffening (8), and impaired diastolic filling (4). Similar preclinical alterations occur across other cardiovascular and cardiometabolic disorders: atrial fibrosis and mitochondrial remodelling may develop before atrial fibrillation (9, 10), endothelial dysfunction precedes hypertension and atherosclerosis (11) and even rare diseases such as J-wave syndromes are now considered cardiomyopathies with subtle structural abnormalities that remain undetected by standard imaging techniques (12). In addition, disturbances in protein homeostasis and metabolic adaptation occur long before the onset of clinical manifestations of the disease (13). Thus, sudden cardiac pathological events are often preceded by manifestations of biological processes that have been unfolding over several years. The temporal trajectories differ markedly across various pathologies (12, 14), are not linear (15), and are modulated by age, sex, lifestyle and inter-organ interactions. This interindividual variability calls for a personalised temporal framework for diagnosis and therapy, rather than a single, universally applied diagnostic threshold. Disease is typically defined once a threshold value is exceeded (4). In HF, this heterogeneity is further reflected in the attempts to subcategorize distinct phenotypes, notably HF with reduced ejection fraction (HFrEF), HF with mildly reduced ejection fraction (HFmrEF) and HF with preserved ejection fraction (HFpEF). This differentiation likely represents overlapping but temporally and mechanistically divergent trajectories rather than strict separate entities (16). Similarly, this limitation is evident in the study and management of AF, where the conventional classification into paroxysmal and persistent forms, subsequently expanded to include long-standing persistent and permanent AF, fails to adequately capture individual risk profiles and the progressive nature of the underlying atrial myopathy (17). Emerging approaches based on biomarkers (18) and wearable or implantable devices (19, 20) will increasingly enable real-time monitoring of individual disease trajectories for earlier detection of warning signals before irreversible remodelling occurs. A central challenge is to identify the therapeutically actionable window during which pathological remodelling remains reversible in order to limit disease progression. This limitation highlights the constraints of traditional frameworks based on universally applied symptomatology, isolated criteria and therefore simple causal relationships that inform therapeutic options (21). Given that cardiovascular (patho)biology emerges from continuous interactions across molecular pathways, cells, tissues, and inter-organ communication (21, 22), cardiovascular diseases can be approached as a dynamic, multi-scale and complex trajectory extending from molecular networks to whole-organ function. However, even apparently linear causal relationships at the organ level are grounded in nonlinear molecular and cellular dynamics, highlighting the need for a multi-scale temporal framework. This trajectory is shaped against the background of biological aging and the dynamics of complex systems. This perspective requires a multidimensional framework in which time is considered an essential dimension of disease. Time is not only a static background information, but through aging it is also an active determinant of adaptation, remodelling, and failure. It should be note that the term “four-dimensional” has previously been used in cardiovascular cronobiology to describe the circadian regulation of cardiac physiology across an hourly-to-daily timescale (23, 24). Here, however, the fourth dimension captures the longitudinal trajectory of disease development and biological aging over years. These represent distinct temporal scales and conceptual frameworks. Here, we argue that incorporating temporal biology into cardiovascular research, from molecular clocks to organ-level remodelling trajectories, is essential to advance precision cardiology and to identify therapeutically relevant temporal windows prior to the point of no return.

## 2 Biological age and the insidious development of disease over time

In medicine, diagnosis is typically established when a physiological parameter deviates from a defined normal range. For example, hypertension is diagnosed when blood pressure exceeds a fixed numerical value, whereas HF is defined once the ejection fraction falls below a critical value (25). HFrEF (EF ≤40%), HFmrEF (EF 40%–49%) and HFpEF (EF ≥50%) represent 3 clinical phenotypes (16) that share common pathophysiological mechanisms, neurohormonal modulation, myocardial fibrosis and inflammation, but differ in their temporal trajectories which may be influenced by major clinical events such as myocardial infarction, as well as in their therapeutic responses. These diagnostic thresholds define disease at the moment of clinical recognition rather than at its biological origin, reinforcing the perception that health ends and disease begins at the time of diagnosis rather than long before the clinical manifestation. This reflects a conceptual limitation: disease is anchored to clinical detection rather than to the underlying biological and time-dependent trajectory. Furthermore, diagnostic thresholds are often based on normal values established in relation to chronological age even though the cardiovascular system may be biologically much older or younger. For instance, the coronary endothelium of a 50-year-old patient may exhibit a molecular signature typically observed in older subjects, characterized by the accumulation of senescent cells and impaired nitric oxide signalling (26). Conversely, an octogenarian with a biologically younger myocardium, characterized by preserved mitochondrial function, low systemic inflammation, and maintained protein homeostasis, may remain healthy beyond the expected clinical limits (27) (Figure 1).

**Figure 1:**
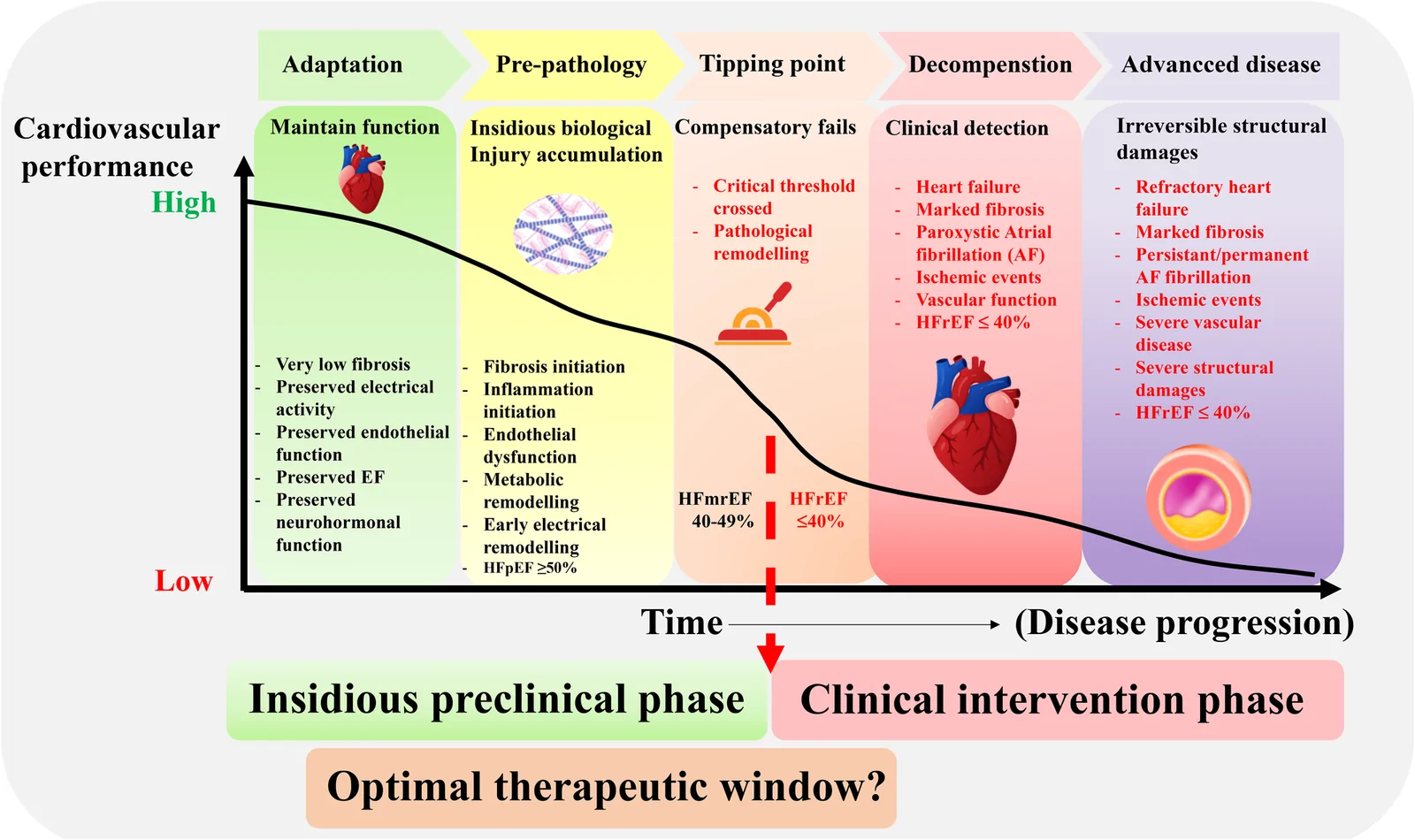
Temporal trajectory of cardiovascular performance across disease progression.

Cardiovascular function declines gradually long before symptoms appear, while the body compensates to maintain normal performance. Insidious damage accumulates over time until these compensatory systems fail. At that point, disease becomes visible, even though the best opportunity for prevention was earlier. Heart failure with reduced ejection fraction (HFrEF), characterized by impaired systolic function and reduced cardiac output, typically showing strong response to guideline-directed medical therapy; heart failure with mildly reduced ejection fraction (HFmrEF), representing an intermediate phenotype between reduced and preserved ejection fraction; heart failure with preserved ejection fraction (HFpEF), characterized by preserved systolic function but impaired ventricular relaxation and increased stiffness, often associated with systemic comorbidities. In the case of the octagenerian, compensatory mechanisms maintain an apparent state of normality even when myocardial hypertrophy may exist for years, together with progressive fibrosis, while preserved cardiac output and endothelial repair transiently sustain cardiac and vascular function. Beneath this clinical silence, biological vulnerability progressively accumulates until compensatory reserves are exhausted. The insidious phase represents a key but systematically underexplored therapeutic window, when intervention may be most effective, but where detection is limited. Indeed, an EF ≤ 40% only reflects vulnerability and biological age at the moment it is measured. The EF values shown correspond to clinically recommended thresholds and illustrate the progression of cardiac dysfunction linked to HF (28). The undiagnosed patient, in whom structural and functional cardiac changes accumulate silently over years, better illustrates this limitation.

## 3 The tipping point problem

Considering the temporal development of cardiovascular disease, several questions arise: at what exact point does a stable system become pathologically unstable? How can long periods of apparent stability suddenly give way and unmask latent failure? And why do individuals exposed to similar stressors exhibit such divergent outcomes? How can we use time-related pathology to identify and potentially treat patients at risk for cardiovascular disease at an early stage? Each disease follows a trajectory that remains largely undetected and is shaped by biological, genetic, proteomic, environmental, and temporal factors. When the resulting, cardiovascular, biological age exceeds chronological age, adaptive capacity is reduced and the threshold for transition is lowered (29). Interestingly, the naked mole-rats maintain cardiac function into their fourth decade of life, demonstrating that biological and chronological aging can be profoundly uncoupled (30). Conversely, biologically younger systems tolerate similar stress without decompensation. Biological systems initially buffer stress through compensatory mechanisms, but once a critical threshold is crossed, they shift rapidly and irreversibly toward a pathological state (31). Disease does not emerge abruptly but it reflects a progressive erosion of adaptive reserve that progresses toward a tipping point (32) [see Figure 1)]. Identifying early signals of this decline, before the system fails, is therefore central to the temporal framework proposed here, and may explain why clinically stable periods can precede sudden and unexpected decompensation (33–35). The progressive loss of adaptive capacity may eventually lead to an abrupt transition to a pathological state, consistent with the concept of critical transitions in complex systems. As a system approaches a critical threshold, critical slowing down and changes in its dynamics, including increased variance and instability, may provide early-warning signals of this transition (36, 37). Applied to cardiovascular disease trajectories, this framework may help identify early, subclinical signs of maladaptive capacity before irreversible decompensation or apparent cardiac disorders. Yet, these signals remain largely invisible to current diagnostic tools, underscoring the need for a new analytical framework. These dynamics differ across HF (or across other pathologic) phenotypes and directly influence the optimal timing of intervention. HFrEF often reaches a tipping point marked by irreversible cardiomyocyte loss and fibrosis, whereas HFpEF may progress more gradually through accumulating diastolic dysfunction and microvascular impairment. HFmrEF may represent an intermediate and potentially more reversible stage. Determining where patients are positioned along this trajectory is crucial for identifying the window during which intervention can still prevent irreversible decompensation. Insight into the “natural history” of the various diseases therefore is of essence.

## 4 Plea for four-dimensional cardiovascular research

Systems biology has transformed cardiovascular research through multi-omics integration, spatial transcriptomics, single-cell sequencing, and computational physiology (38, 39). However, these advances remain incomplete without explicit integration of time and multimodality tailored to the pathology under study. Indeed, most experimental models rely on young male animals, despite growing attention to include both sexes in experimental practice (40), and fail to recapitulate human biological aging and cumulative environmental exposures (smoking, diet, inactivity, obesity) (41),. One example is the cumulative electrophysiological remodelling that develops over years (42). This process is not limited to a time-dependent prolongation of action potential duration, but rather reflects a gradual disruption of ion-channel homeostasis, mechano-electrical coupling, and repolarisation reserve that grows with biological aging and our environment. The biological mechanisms are often investigated in isolation, despite their strong temporal interdependence (21, 43). Similarly, many experimental disease models may bypass the slow intermediate stages that are central to human cardiovascular aging. This progressive remodelling is associated with QT interval prolongation and increased repolarisation heterogeneity on the ECG, reflecting a growing ionic imbalance and destabilisation of the spatial and regional electrophysiological control systems. Collectively, these changes increase susceptibility to ventricular arrhythmia and electrical instability in an age-dependent manner (30, 44–46). Thus, current cardiovascular research remains largely reductionist and linear (1-dimensional). Some approaches integrate aspects of cardiac function but neglect systemic interactions (2 dimensional); Some recognise the interdependent networks across molecular, cellular, tissue, and inter-organ levels (21) (3-dimensional). In this context, a 4-dimensional framework incorporates time as an active dimension, capturing disease as a trajectory through adaptive, pre-pathological, unstable, and decompensated states, and revealing therapeutically relevant windows before the tipping point is reached. However, the validity of such a framework depends, in part, on the experimental models used to study these dynamics and here a critical limitation emerges. Most of the AF/HF-related models are induced in young animals, which lack the cumulative electrophysiological substrate, including age-related calcium dysregulation, oxidative stress, fibrosis and reduced repolarisation reserve that in combination underlie arrhythmogenesis in humans. As a result, such models poorly recapitulate, or fail to reproduce, transient arrhythmogenic events, including for example early and delayed afterdepolarisations at the cellular level. The reconstruction of true cardiac disease dynamics therefore remains fundamentally and always limited. A central challenge is the integration of biological age as a mechanistic dimension of disease. Rather than being treated as a simple time-based variable, biological age (47) should reflect molecular damage, immune remodelling, mitochondrial dysfunction, and ion-channel alterations. Epigenetic clocks such as GrimAge (48) and PhenoAge (49) enable estimation of biological age independently of chronological age. Integrating these measures into digital twin frameworks would allow reconstruction of disease trajectories and quantification of the velocity of aging over time, distinguishing adaptive, pre-pathological, unstable, and decompensated states and capturing transitions rather than static categories. Together, structural remodelling, electrophysiological instability, inflammatory signalling, and metabolic aging likely interact synergistically to reduce cardiovascular adaptive capacity over time (Table 1). Altogether, they call for a paradigm shift toward a 4-dimensional cardiovascular biology in which time is not a background variable but a central dimension of disease structure and progression. Four-dimensional modelling is particularly relevant for distinguishing HFrEF, HFmrEF and HFpEF as temporally divergent trajectories. Longitudinal integration of electrophysiological, structural and metabolic biomarkers may reveal the dynamic transition points between these 3 phenotypes and identify personalised intervention windows.

**Table 1:** Temporal phases of cardiovascular performance and their clinical implications.

Temporal phase	Biological features	Detectable signals	Clinical implications
1. ADAPTATION (Compensated)	Very low fibrosis, preserved electrical activity & endothelial function, preserved EF & neurohormonal function	Subtle changes in BNP, slight elevation in hs-CRP, minor diastolic dysfunction on echocardiography	Early window for lifestyle and pharmacologic interventions; high potential for reversibility
2. PRE-PATHOLOGY (Insidious injury)	Fibrosis & inflammation initiation, endothelial dysfunction, metabolic remodelling, early electrical activity, HFpEF ≥50%	Elevated galectin-3, subtle ECG changes, altered heart rate variability, early diastolic filling abnormalities	Opportunity for targeted prevention; risk stratification using biomarkers and imaging
3. TIPPING POINT (Compensatory fails)	Critical threshold crossing & pathological remodelling, balance between HFmrEF 40–49% & HFrEF≤40%	Paroxysmal AF, acute decompensated HF, first MI	Rapid transition to overt disease; often irreversible without aggressive intervention
4. DECOMPENSATION (Clinical detection)	Heart failure, Paroxystic AF, ischemic events, Marked fibrosis & vascular dysfunction, HFrEF≤40%	Clinical HF symptoms, persistent AF on ECG, recurrent angina & stroke	Limited reversibility; focus shifts to symptom control, risk reduction, and device/ablative strategies
5. ADVANCED DISEASE (Irreversible structural damages)	Refractory heart failure, Persitant/permanent AF, severe ischemic events, severe vascular dysfunction, marked fibrosis & severe structural damages, HFrEF≤40%	Clinical HF symptoms, persistent/permanent AF on ECG, recurrent angina & stroke	Limited reversibility; focus shifts to symptom control, risk reduction, and device/ablative strategies

Heart failure with reduced ejection fraction (HFrEF), characterized by impaired systolic function and reduced cardiac output, typically showing strong response to guideline-directed medical therapy; heart failure with mildly reduced ejection fraction (HFmrEF), representing an intermediate phenotype between reduced and preserved ejection fraction;, heart failure with preserved ejection fraction (HFpEF), characterized by preserved systolic function but impaired ventricular relaxation and increased stiffness, often associated with systemic comorbidities.

## 5 Biological time inversion: myth or reality?

Reprogramming strategies may hold potential across HF phenotypes. In HFpEF, where early remodelling remains mainly metabolic and epigenetic rather than structural, biological time modulation could theoretically restore adaptive capacity. The degree of functional recovery in advanced HFrEF is probably limited by irreversible cardiomyocyte loss. The idea of biological time inversion, which suggests that some features of aging may be completely or partially reversible under controlled experimental settings, is still controversial. Partial *in vivo* reprogramming through transient Yamanaka factor (OSKM) expression has been shown to reset epigenetic landscapes while preserving cellular identity, restoring chromatin accessibility and regenerative capacity in aged tissues (50, 51). Naked mole rats may reach an age of 40 years with remarkably limited cardiovascular aging (30). Equally intriguing, a study using heterochronic parabiosis models indicate that exposure to a young systemic milieu can reverse cardiac hypertrophy and improve myocardial function in aged mice (52), although the identity and reproducibility of circulating rejuvenating factors remain controversial, particularly for candidates such as GDF11 (53, 54). More recently, partial reprogramming to neural and sensory systems, involving restoration of vision in aged mice, supporting the concept of persistent epigenetic plasticity in adult mammalian tissues (51). Collectively, these findings challenge the classical view of aging as a strictly linear and irreversible trajectory, suggesting instead a dynamic continuum of modifiable biological states. However, the magnitude, durability, and safety of such (therapeutic) effects remain highly context-dependent across tissues and experimental platforms, with unresolved risks including tumorigenicity, loss of cellular identity, and long-term genomic instability. In the cardiovascular system, where remodelling emerges from tightly integrated mechanical, metabolic, and inflammatory signalling, such plasticity appears partial and temporally constrained. Importantly, these observations reinforce the concept that cardiac aging and disease progression are not fixed endpoints but time-dependent processes shaped by reversible and irreversible phases and by parallel temporal dynamics across organs and cell systems. This framework directly applies to the temporal model of cardiovascular disease proposed above, in which early remodelling may remain accessible to intervention before the system reaches its tipping point. Accordingly, biological time modulation should be viewed not as a true reversal of aging, but as a constrained reprogramming of cardiovascular trajectories within a broader 4-dimensional disease continuum. However, the translation of such approaches to human cardiovascular disease remains limited by concerns over safety, stability, and long-term functional integration issues.

## 6 Conclusion

Cardiovascular diseases rarely begin at symptom onset. When HF, AF, or coronary disease becomes clinically apparent, the underlying biology has often been active for years. The observed pathology reflects the final stage of a prolonged silent process. A central feature of this process is the divergence between biological and chronological (cardiovascular) age, which shapes the rate of progression and proximity to the tipping point. This mismatch influences risk, reversibility, and therapeutic timing. Recognising that the individual cardiovascular system may be biologically older or younger than its chronological age represents a mechanistic determinant with implications for prevention and clinical intervention. Redefining HFrEF, HFmrEF and HFpEF, as well as paroxysmal and persistent AF, through a temporal and biological age framework transforms them from diagnostic labels into dynamic disease states. This shift allows interventions to be timed to an individual's progression toward phenotype-specific tipping points, rather than to symptom onset. The next paradigm shift in cardiovascular research will be fundamentally conceptual. It will require integration of the principles of personalised medicine and moving from static, symptom-driven definitions of disease toward dynamic, trajectory-based frameworks grounded in systems biology. The focus will shift from detecting electromechanical/structural abnormalities to unveiling early signs of instability across different levels of biological organisation. Without integrating biological time as a fourth dimension, cardiovascular biology remains fundamentally incomplete, and progress toward improved patient outcomes will continue to be constrained by the lack of more integrative and translational research frameworks. The recognition that cardiovascular disease processes begin long before clinical manifestations provides a rationale for incorporating biological time into our understanding of disease progression. This perspective may help identify early transitions and therapeutic windows before irreversible decompensation and apparent disease. It also suggests that causality in cardiovascular disease should be understood in direct relation to biological trajectories and their evolution over time, rather than solely by the timing of events.
